# Factors influencing cost awareness in hospitals: a scoping review

**DOI:** 10.1186/s12913-025-13476-0

**Published:** 2025-12-05

**Authors:** Nikma Fitriasari, Heri Prayitno, Diah Ayu Puspandari, Andreasta Meliala, Adi Utarini

**Affiliations:** 1 Medical and Health Sciences, Faculty of Medicine, Public Health, and Nursing, Universitas Gadjah Mada, Yogyakarta, Indonesia; 2 Department of Public Health, Public Health Faculty, Universitas Brawijaya, Malang, Indonesia; 3 Brawijaya University Library, Universitas Brawijaya, Malang, Indonesia; 4 Department of Health Policy and Management, Faculty of Medicine, Public Health, and Nursing, Universitas Gadjah Mada, Yogyakarta, Indonesia

**Keywords:** Cost awareness, Cost consciousness, Hospital, Healthcare

## Abstract

**Background:**

Financing healthcare services remains a significant challenge in developing countries, with a 30% increase in healthcare costs attributed to the overutilization of diagnostic tests and therapies. Low-cost awareness within the hospital setting may contribute to this situation. This scoping review aims to identify factors influencing cost awareness and explore strategies for enhancing cost awareness in hospitals.

**Methods:**

The scoping review method followed the framework of Arksey and O’Malley. Eligibility criteria used the Population, Concept, and Context mnemonic. The population covers all hospital workers with the concept of factors influencing cost awareness and strategies to enhance. The context encompasses hospitals across all countries. Literature was searched in seven databases (EBSCO, Cochrane Library, ProQuest, ScienceDirect, Scopus, PubMed, Web of Science), and through hand searching and grey literature. Data were extracted from publications between January 1990 to March 2024, coded inductively, and categorized thematically.

**Results:**

From 984 identified records, 54 studies met the inclusion criteria. Most were conducted in the United States (53.7%) and Europe (25.9%), with limited representation Asia (12.9%) and Africa (5.6%). Quantitative cross-sectional design was the most common methodology (40 studies, 74.1%), followed by intervention studies (11 studies, 20.3%) and qualitative studies (3 studies, 5.6%). Intervention studies were predominantly implemented in the United States, with one conducted in Europe. Internal factors, particularly professional knowledge (51.8%) and experience (42.6%), were the most frequently cited determinants. Organizational factors such as access to price information and cost containment policies were also influential. External factors included patient expectations, healthcare financing policies, and academic curricula. Cost education and visual pricing systems emerged as the most frequently cited strategies to improve cost efficiency and healthcare outcomes. Interventions were predominantly implemented in teaching hospitals in high-income countries.

**Conclusions:**

Cost awareness in hospitals is shaped by internal and external factors, with professional knowledge, experience, and access to price information emerging as common influences. Cost education and visual pricing systems were the most frequently reported with evidence mostly from the high-income settings. Future research, particularly in low- and middle-income countries, should focus on developing and testing context-specific, multisectoral strategies to strengthen cost awareness across diverse hospital settings.

**Trial registration:**

This scoping review is registered with the Open Science Framework at https://osf.io/7h5et /(OSF | Factors influencing cost awareness in hospitals: a scoping review).

**Supplementary information:**

The online version contains supplementary material available at10.1186/s12913-025-13476-0.

## Introduction

Financing healthcare services remains a critical issue, particularly in developing countries [1], where healthcare financing has been outpacing global economic growth, comprising 10% of the global gross domestic product (GDP) [2, 3]. This trend is driven by multiple factors [4], including changes in the disease patterns such as occurred during the global economic crisis of 2008–2012 and the COVID-19 pandemic since 2020 [5, 6].

Escalating healthcare expenditures remains a global issue, largely due to weak cost containment measures and regulatory oversight [7]. In the United States, the “law of medicine money” illustrates how healthcare spending tends to absorb available resources as the inherently consumptive nature of delivering optimal care continues to elevate costs for both patients and providers [8, 9].

Cost of hospital care is most pronounced, i.e., 32.1% of total healthcare expenditures, exceeding other healthcare sectors [8, 10, 11]. As a result, hospitals are central to cost containment efforts. This consumptive nature, including overuse of diagnostic tests and therapies contributes to up to 30% of excess healthcare expenses [12–15]. Studies estimate that 30–50% of diagnostic tests are unnecessary, leading to substantial waste [13, 16]. With treatment decisions accounting for 80–87% of healthcare service costs [14, 17], cost awareness among doctors became crucial.

Poor cost awareness among doctors have been demonstrated from several studies [12, 18–20], suggesting the need to enhance cost awareness aim to control costs, optimize resource utilization, and eventually achieve cost efficiency and effectiveness [17]. Decreased resource utilization while maximizing health benefits with the available funds collectively contribute to a 60% reduction in healthcare costs in hospitals [21, 22].

Enhancing cost efficiency in hospital care requires programs that promote cost awareness among the healthcare workers [15, 23]. However, a lack of understanding of the factors influencing cost awareness limits the development of effective strategies. Despite ongoing efforts, awareness remains low, particularly in hospital settings, thus contributing to inefficiencies such as overuse of diagnostic tests and therapies [24]. Studies consistently link unnecessary diagnostic testing to escalating healthcare expenditures, highlighting the need for more judicious resource utilization to improve care quality and reduce financial burden [25].

Alongside promoting cost awareness, strategic provider payment models can improve efficiency and cost control by influencing healthcare providers behaviors. Alternative payment methods such as pay-for-performance (P4P) and bundled payments, align financial incentives with high-quality of care [26]. P4P ties compensation to performance metrics, encouraging efficient resource use and better patient outcomes [27, 28], while bundled payments provide a single payment for all services related to a treatment, discouraging unnecessary procedures and fostering care coordination [27, 29]. Adoption of these methods support both cost awareness and broader cost-containment strategies.

This scoping review seeks to bridge this critical knowledge gap to improve cost awareness in hospital setting. The method organizes and synthesizes evidence to provide holistic conceptual clarity [30], enabling the systematic identification of factors influencing cost awareness and the development of tailored strategies for hospital environments. This study aims to identify the factors influencing cost awareness among all hospital staff and to highlight the cost awareness strategies implemented in hospital settings.

## Method

### Design

The scoping review method was selected due to its ability to encompass a broad topic by synthesizing various studies with different designs-methodologies [30] and the objective of this review is to map and summarize the available literature, particularly in areas where the evidence is sparse or emerging [31]. Compared to the previous review on cost awareness, i.e. two systematic reviews published (2007, 2008) and one scoping review (2024), this review involves all hospital workers and covers updated publications up to March 2024. Therefore, this expanded scoping review is expected to provide strategies informed by the latest publications on cost awareness among diverse hospital workers.

This scoping review follows the framework established by Arksey and O’Malley, with recent refinements by Levac et al. [32, 33]. These six stages of this framework involved [1]: identifying the research question [2], identifying relevant studies [3], selecting articles [4], extracting data [5], organizing, summarizing, and reporting findings, and [6] consultation [31]. As consultation with stakeholders is not mandatory in scoping reviews [34], this step was not conducted. The scoping review was registered with the Open Science Framework at https://osf.io/jt4u7/(OSF | Factors influencing cost awareness in hospitals: a scoping review).

### Eligibility criteria

The scoping review’s identification of relevant studies followed the Population, Concept, and Context (PCC) mnemonic outlined in Table 1 [35]. The chosen population encompassed all hospital workers, allowing for an examination of cost awareness levels among medical, paramedical, and non-medical staff. The primary focus of study selection is around factors influencing cost awareness, with additional attention given to interventions or strategies aimed at its development. The review context encompassed hospitals across all countries. Selected studies for synthesis included empirical research written in English, using various research designs, both quantitative and qualitative. Studies involving patient populations, insurance, government, medical students (clerks and co-assistants), and family doctors were excluded. Additionally, studies focusing on value care, economic evaluation, clinical settings, and private practice doctors were not included.

**Table 1:** Eligibility criteria

Criteria	Population	Concept	Context	Data Source Type
Inclusion	Healthcare workers	1. Factors influencing cost awareness	1. Hospitals	1. English language
		2. Intervention or strategy to create cost awareness	2. All countries	2. Empirical study (All types of study design) 3. Grey literature (theses and dissertations)
**Exclusion**	1. Patients	1. Value care	1. Clinics	1. Editorials
	2. Insurance 3. Government 4. Medical students 5. Family doctors	2. Economic evaluation	2. Private practice doctors	2. Letters to the editor 3. Narrative comments 4. Webpages 5. Government policies

### Information sources

Publications were selected from seven electronic databases covering various disciplines relevant to the topic of cost awareness: EBSCO, Cochrane Library, ProQuest, ScienceDirect, Scopus, PubMed, and Web of Science. The search was conducted on March 20–22, 2024 and publications from January 1990 to March 2024 were identified. Additionally, a supplementary hand-searching process was conducted to ensure a comprehensive scoping review. This involved screening the reference lists of three previously published systematic reviews that are aligned with the study’s inclusion criteria. Articles cited in these reviews were systematically searched using Google Scholar, resulting in the identification of four additional relevant studies. Furthermore, to incorporate grey literature, a targeted search for theses and dissertations related to cost awareness was performed via Google Scholar. This approach yielded two master’s theses and one doctoral dissertation. Given that Google Scholar does not allow filtering by title and abstract, the search strategy was refined by using specific keywords in combination with Boolean operators and reviewing the full text of potentially relevant documents.

### Search strategy

Using the Parsifal tool, potential keywords were identified through discussions between researchers and consultations with the Librarian of Universitas Brawijaya Library [36, 37]. Parsifal aids researchers in conducting systematic reviews by suggesting potential keywords. Using the search feature within the Scopus and ScienceDirect databases, the librarian could identify relevant publications and evaluate them to determine potential keywords. The search strategies employed across the seven databases and Google Scholar are outlined in Table 2.

**Table 2:** Search keywords and the queries used for factors influencing cost awareness

Databases	Keywords and queries
EBSCO	**Keywords**: healthcare workers, medical staff, medical personnel, internal staff, house staff, cost awareness, cost consciousness, healthcare services, healthcare facilities, healthcare providers, medical facilities **Query**: (“healthcare workers” OR “medical staff” OR “medical personnel” OR”internal staff” OR “house staff”) AND (“cost awareness” OR “cost consciousness”) AND (”Hospital OR Healthcare services“OR “healthcare facilities” OR “healthcare providers” OR “medical facilities”)
Cochrane Library	**Keywords**: cost awareness, cost consciousness, hospital, healthcare **Query**: ((“cost awareness” OR “cost consciousness”)): ti, ab, kw AND ((hospital OR healthcare)): ti, ab, kw
Proquest	**Keywords**: cost awareness, cost consciousness, hospital, healthcare **Query**: (“cost awareness” OR “cost consciousness”) AND (hospital OR healthcare)
Science direct	**Keywords**: cost awareness, cost consciousness, hospital, healthcare **Query**: (“cost awareness” OR “cost consciousness”) AND (hospital OR healthcare)
Scopus	**Keywords**: cost awareness, cost consciousness, hospital, healthcare **Query**: TITLE-ABS-KEY ((“cost awareness” OR “cost consciousness”) AND (hospital OR healthcare)) AND (LIMIT TO (PUBSTAGE , “final”)) AND (LIMIT-TO (DOCTYPE , “ar”)) AND (LIMIT-TO (LANGUAGE , “English”)) AND (LIMIT-TO (SRCTYPE , “j”)) AND (LIMIT-TO (OA , “all”))
PubMed	**Keywords**: cost awareness, cost consciousness, hospital, healthcare **Query**: (“cost awareness” OR “cost consciousness”) AND (hospital OR healthcare)
Web of Science	**Keywords**: cost awareness, cost consciousness, hospital, healthcare **Query**: (“cost awareness” OR “cost consciousness”) AND (hospital OR healthcare)
Google Scholar	**Keywords**: cost awareness, cost consciousness, hospital, healthcare **Query**: (“cost awareness” OR “cost consciousness”) AND (hospital OR healthcare)

### Selection of evidence sources

All identified studies were imported into Parsifal, a web-based tool that facilitates planning, conducting, and documenting systematic reviews through form completion. Parsifal offers support for searching Scopus and ScienceDirect publication databases directly from the tool. It also enables detailed monitoring of systematic reviews and allows researchers to facilitate initial screening of titles and abstracts, as well as collaboration among reviewers [37].

To establish eligibility criteria, the reviewer team (NF, HP, DA, AM, AU) underwent a two-stage process to reach a consensus. Initially, two reviewers (NF, DA) tested the criteria on 20 sample abstracts, addressing any inconsistencies and clarifying criteria as needed. Subsequently, all five reviewers evaluated the established criteria, resulting in a consensus with minor improvements [35, 38].

Study selection involved two reviewers (NF, DA) in two stages. Initially, studies were screened based on title and abstract using predefined inclusion and exclusion criteria, categorizing them as “accepted,” “rejected,” or “unclassified.” Conflicting decisions were resolved through consensus discussions among reviewers. In the second stage, selected abstracts were independently screened in full text, with any conflicting decisions addressed through further discussion within the reviewer team until a consensus was reached.

### Data charting and data items

The data extraction form was collaboratively developed by the reviewer team. Data extraction involved capturing both study characteristics and research findings [39]. The following study characteristics were extracted: author names, publication year, study location, service setting, study design, and respondent. The research findings were extracted based on data describing the factors influencing cost awareness and recommended strategies for enhancing cost awareness. Following the JBI and PRISMA-ScR guidelines for scoping reviews, a formal quality appraisal of the included studies was not conducted.

### Summary of results

The primary aim of the scoping review was to map research results and summarize research findings [30]. Achieving this objective necessitated content analysis. Despite the limited knowledge of factors influencing cost awareness, an inductive approach to analysis was considered appropriate [31]. Inductive analysis consisted of identifying, coding, and categorizing information. Identifying involves identifying recurring ideas, concepts, phrases, or patterns within the data. Coding was conducted by assigning the same numerical label to similar information content, followed by categorizing, i.e. grouping similar codes into a category. The coding and categorization framework underwent review by all members of the review team [40]. Reporting of the scoping review adhered to the Preferred Reporting Items for Systematic Reviews and Meta-Analyses extension for Scoping Reviews (PRISMA-ScR) checklist [41, 42].

## Results

### Study selection

**Fig. 1:**
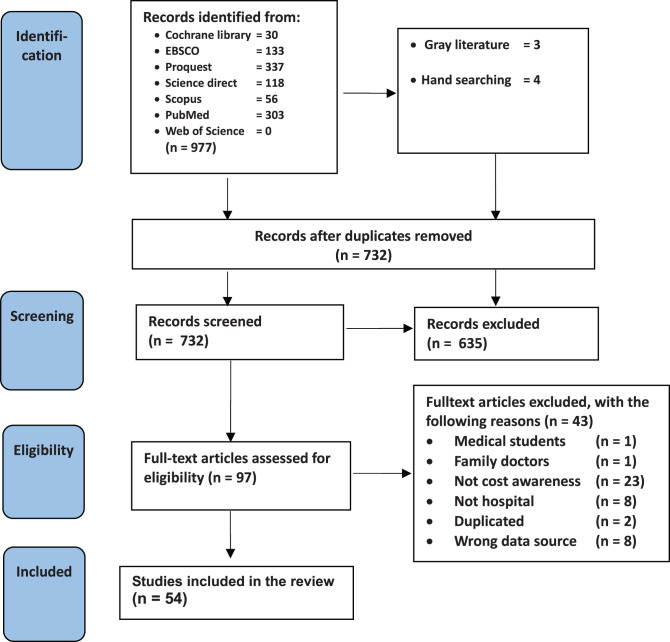
PRISMA flow diagram

Searches across seven databases yielded 977 studies (Fig. 1). Additionally, three documents from grey literature sources (i.e. two theses and one dissertation) and four studies through hand searching were obtained, contributing to a total of 984 studies. After the removal of duplicates through screening of the title and abstract, 732 studies remained. Subsequently, 97 studies were identified for full-text screening, resulting in the remaining 54 studies.

### Study characteristics

Study characteristics and research findings were presented for 54 studies (Table 3). Most research was conducted in the US (29 studies or 53,7%), and the rest was in Europe (14 studies or 25,9%), Asia (7 studies or 12,9%), Africa (3 studies or 5,6%), and multi-countries (1 studies or 1,8%). Cost awareness studies were primarily conducted in teaching hospital settings (27 studies or 50%), particularly in emergency, surgical, intensive care, anesthesia, internal medicine, obstetrics and gynecology, and pediatric departments, with doctors comprising most respondents. Quantitative research with a cross-sectional design was the most common methodology (40 studies or 74,1%) and 11 studies (20,3%) applied intervention design (quasi-experimental and randomized-controlled trial). All intervention studies were carried out in America with one study conducted in Europe. Less common design was qualitative research (3 studies or 5,6%).

### Factors influencing cost awareness

Table 4 outlines two categories of factors influencing cost awareness, namely internal and external factors.

**Table 3:** Data extraction

Author (s)/ Year/ Cite	Publication characteristics		Study design & Participant	Cost awareness factors						Cost awareness strategy			
	Location	Setting		Internal factors		External factors				Human resources	Organizational	Technology	External relationship
				Individual	Organizational	Key trend	Market	Industry	Academic				
Tierney et al, 1990, [43]	America	Teaching Hospital (Laboratory)	RCT (doctors)		v							v	
Manheim et al, 1990, [44]	America	Teaching Hospital (Emergency)	RCT (doctors)	v						v			
Horrow et al, 1994, [45]	America	All hospital type (General setting)	RCT (doctors, nurses)	v	v		v			v		v	
Santosa et al, 1997, [46]	Asia	Private Hospital (General setting)	Cross-sectional (managers)						v	v			
Conti et al, 1998, [47]	Europe	Teaching Hospital (Intensive care)	Cross-sectional (doctors)	v	v	v				v		v	v
Lin et al, 1998, [48]	America	Teaching Hospital (Anesthesia)	Quasy-experimental (doctors)							v	v	v	
Amtssygehus, 1999, [49]	Europe	Teaching Hospital (Anesthesia)	Cross-sectional (nurses, doctors)	v	v					v	v		
Innes et al, 2000, [21]	America	All hospital type (Emergency)	Cross-sectional (doctors)	v	v		v	v		v		v	
Bovier et al, 2005, [53]	Europe	All hospital type (General setting)	Cross-sectional (doctors)	v	v					v	v		
Miron et al, 2008, [54]	Asia	All hospital type (General setting)	Cross-sectional (doctors)	v	v					v	v	v	
Wax et al, 2009, [55]	America	Teaching Hospital (Anesthesia)	Cross-sectional (doctors, nurses)	v						v	v	v	
Paruntu, 2012, [77]	Asia	Public Hospital (Radiology)	Qualitative (Managers)							v			
Portale et al, 2013, [78]	America	Teaching Hospital (Emergency)	Cross-sectional (doctors)	v			v			v		v	
Eriksen et al, 2013, [28]	Europe	All hospital type (General setting)	Cross-sectional (doctors)	v						v		v	v
Rohman et al, 2014, [29]	Europe	Teaching Hospital (Surgery)	Cross-sectional (doctors)	v			v	v		v			v
Pourahangarian et al, 2014, [56]	Asia	Teaching Hospital (Internal medicine, Surgery, Obstetrics gynecology, Pediatrics, emergency)	Cross-sectional (doctors)	v	v					v		v	
Sabbatini et al, 2014, [27]	America	All hospital type (General setting)	Qualitative (doctors)	v	v	v	v	v		v	v		v
Hollifield et al, 2014, [50]	America	Teaching Hospital (Emergency)	Cross-sectional (doctors)	v						v	v	v	
Velasquez, 2015, [62]	Europe	All hospital type (General setting)	Qualitative (managers)	v	v	v							
Ryskina et al, 2015, [79]	America	All hospital type (General setting)	Cross-sectional (doctors)		v								
Jakovljevic et al, 2016, [57]	Europe	All hospital type (General setting)	Cross-sectional (doctors, dentists, pharmacists)	v						v			v
Jackson et al, 2016, [80]	America	Teaching Hospital (Surgery)	Cross-sectional (doctors)		v					v			v
Long et al, 2016, [81]	America	Teaching Hospital (Emergency, Internal medicine, Obstetrics gynecology, Pediatric, Orthopedic surgery)	Cross-sectional (doctors)						v			v	v
Zamil, 2016 [63]	Asia	Teaching Hospital (Surgery)	Cross-sectional (doctors)	v	v					v	v		
Gandhi et al, 2017, [64]	America	Teaching Hospital (General setting)	Cross-sectional (doctors)	v	v					v		v	v
Nethathe et al, 2017, [87]	Africa	Teaching Hospital (Anesthesia, Surgery, Intensive care, Obstetrics gynecology)	Cross-sectional (doctors)							v		v	
Hunderfund et al, 2018, [65]	America	All hospital type (General setting)	Cross-sectional (doctors, medical students)	v		v				v		v	
Ryan et al, 2018, [22]	Europe	All hospital type (Surgery)	Cross-sectional (doctors)	v						v	v		
Edwards et al, 2018, [88]	America	Teaching Hospital (Obstetrics gynecology)	RCT (doctors)							v			
Gabarre et al, 2019, [86]	Europe	Public Hospital (Intensive care)	Cross-sectional (doctors, medical students, paramedical staff)	v						v		v	
Schmitz et al, 2019, [83]	Europe	All hospital type (Neonatal intensive care)	Cross-sectional (doctors, nurses)	v	v					v		v	v
Schmidt et al, 2019, [66]	America	Teaching Hospital (General setting)	Cross-sectional (doctors)	v						v		v	
Pei et al, 2019, [82]	America	Teaching Hospital (Surgery)	Quasy-experimental (doctors)							v	v	v	v
Ross et al, 2019, [58]	America	Private Hospital (Obstetrics gynecology)	Quasy-experimental (doctors)	v	v						v		
Poveya et al, 2019, [67]	Europe	Public Hospital, Teaching hospital (surgery)	Cross-sectional (doctors)	v						v	v		
Mordang et al, 2020, [17]	Europe	All hospital type (General setting)	Cross-sectional (doctors, administrative staff, patients)	v	v	v				v			
Dyrbye et al, 2020, [59]	America	All hospital type (General setting)	Cross-sectional (doctors)	v	v					v		v	
Sorber et al, 2020, [68]	America	Teaching Hospital (Surgery)	Cross-sectional (doctors, nurses)	v	v					v		v	
Fadare et al, 2020, [84]	Africa	Teaching Hospital (Pharmacy)	Cross-sectional (doctors)		v	v	v		v		v	v	v
Yip et al, 2020, [69]	America	Teaching Hospital (Paediatric)	RCT (Doctors and medical students)	v	v					v		v	
Wagenberg et al, 2020, [70]	Europe	Teaching Hospital (Intensive care)	Quasy-experimental (doctors)	v						v			
Qin, 2020, [51]	America	Teaching Hospital (Anaesthesy)	Cross-sectional (nurses, doctors)	v	v				v	v	v	v	
Sheckter et al, 2021, [60]	America	Private Hospital (Surgery)	Cross-sectional (doctors)	v	v		v			v			v
GardeziI et al, 2021, [71]	America	Teaching Hospital (Surgery)	Cross-sectional (doctors, nurses)	v	v						v	v	
Mrwetyana et al, 2021, [72]	Africa	Teaching Hospital (Radiology)	Cross-sectional (doctors)	v						v	v	v	
Lane et al, 2022, [2]	America	Public Hospital (Emergency)	Quasi-experimental (doctors, nurses)	v							v	v	
Fabes et al, 2022, [19]	Europe, America, Australia, Oseanisa	All hospital type (General setting)	Cross-sectional (doctors, medical students)	v	v		v			v		v	
Yasin et al, 2022, [85]	Asia	Public Hospital, Private Hospital (General setting)	Cross-sectional (doctors)	v						v			v
Liang et al, 2022, [73]	Asia	All hospital type (General setting)	Cross-sectional (doctors, nurses)	v						v	v	v	
Heiman et al, 2022, [74]	America	Teaching Hospital (Surgery)	Cross-sectional (doctors, nurses)		v								
Dayan et al, 2022, [75]	America	Teaching Hospital (Surgery)	RCT (doctors, nurses, staff)	v								v	
Resnicow, 2022, [52]	America	All hospital type (General setting)	Cross-sectional (doctors)	v			v			v	v		v
Lee et al, 2023, [76]	America	Private Hospital (Laboratory, Pharmacy, Radiology)	Cross-sectional (doctors)	v									v
Ludemann et al, 2024 [61]	Europe	All hospital type (General setting)	Cross-sectional (doctors and nurses)	v						v			
		**Total**		42	26	6	9	3	4	44	19	30	15
		**Percentage (%)**		77,8	48,2	11,1	16,7	5,6	7,4	81,2	35,2	55,6	27,8

**Table 4:** Identified factors influencing cost awareness (*n* = 54)

Factor			Cites	Number of studies (total *n* = 54)	% of the total number of studies	
A. Internal factor	1. Individual factor	a. Professional characteristic	[23, 43–52]	11		20,3
		b. Job type	[19, 22, 52–61]	12		22,2
		c. Professional knowledge	[2, 17, 21, 22, 44, 45, 47, 49–51, 54, 56, 61–76]	28		51,8
		d. Professional experience	[2,17,19, 22,34,47,49, 52–54,57,64,66,69,72,76–82]	23		42,6
		e. Job satisfaction	[27, 45, 47, 49, 53, 59–62, 65, 73, 75, 78, 83]	13		24,1
	2. Organizational factor	a. Price information access	[21, 27, 43, 45, 47, 49, 51, 56, 64, 68, 69, 71, 80, 84]	14		25,9
		b. Hospital type	[53, 58, 83]	3		5,6
		c. Cost containment policy	[19, 49, 54, 62, 63, 68, 79]	7		12,9
		d. Workplace Culture	[17, 71, 74, 79]	4		7,4
		e. Physical factor	[19, 59, 60]	3		5,6
B. External factor	1. Key trend factor	a. Health financing policy	[17, 27, 47, 62, 65, 84]	6		11,1
	2. Market factor	a. Patient	[19, 21, 27, 29, 45, 52, 60, 84]	8		14,8
	3. Industry factor	a. Supplier	[21, 27, 29]	3		5,6
	4. Academic factor	a. Academic curriculum	[46, 51, 81, 84]	4		7,4

### Internal factors

Internal factors were the most reported factors affecting cost awareness (46 studies or 85,2%), consisting of individual factors (reported in 42 studies or 78%) and organizational factors (26 studies or 48%).

Among the individual factors, professional knowledge and experience emerged as critical influences. Professional knowledge on costs was low [2, 21, 22, 45, 47, 49, 50, 63–65, 67, 68]. This includes knowledge about drug costs [28, 45], medical equipment [47, 51, 54], and diagnostic tests [78]. Professional experience plays a pivotal role in shaping cost awareness and decision-making among doctors, influencing their approach to patient care [60] and resource utilization [45, 83]. Less reported internal factors were job satisfaction, job type, and professional characteristics. Job satisfaction varies across professional status, playing a crucial role in shaping work habits and the degree of attention paid to cost-effective practices [49, 75]. Likewise, professional characteristics such as age [60, 85], gender [53], education level [55, 73, 86], and race/ethnicity [60] were also important.

Regarding organizational factors, price information access was mostly mentioned, followed by cost containment policies, workplace culture, hospital type, and physical factors. Limited access to price information is often due to a lack of transparency in drug prices and the confidentiality of hospital purchase prices from suppliers [51, 71]. Procurement [46, 68] and budgeting practices involving employees [46, 49, 62], play a crucial role as part of the cost containment policies [79]. A culture of seniority often discourages discussions regarding the utilization of tools and materials in healthcare services, resulting in a lack of transparency regarding the costs of tools and materials in healthcare services [71]. Doctors working in teaching hospitals and public hospitals generally have better cost awareness [83]. Physical factors such as geographical and working location influence cost awareness, with doctors in maritime countries such as Australia and New Zealand showing higher awareness than those in the U.S. [19]. While there is no differences in cost awareness between rural and urban areas [60], burnout in primary care may reduce awareness [59].

### External factors

External factors were mentioned by 16 studies (30%) and encompassed key trends, market, industry, and academic influences. Patients play a key role as market factors, with their expectations [45] and financial behavior [84] significantly shaping cost awareness and decision-making processes [27]. Healthcare financing policies, such as health resource utilization control policies [27], DRG (diagnosis-related group) financing systems [47], and value-based care policies [65], also have considerable influence on cost awareness within hospitals. Integration of cost education during undergraduate and postgraduate studies and ongoing education improved cost awareness [51, 81, 84], unlike the presence of suppliers of drugs, equipment, and healthcare materials [21].

### Strategy for cost awareness development

The strategy for developing cost awareness is categorized based on the innovation cost management theory [46], which consists four strategies: human resources, organizational, technological, and external relations strategies (Table 5). The most frequently used strategies for enhancing cost awareness were human resource (44 studies or 81%), followed by technology (30 studies or 55.6%), organizational (19 studies or 35,2%), and external relationship (15 studies or 27,8%). Among these strategies, RCT design was used to study cost education interventions categorized under human resource strategies [44, 69, 88] and visual pricing strategies [43, 45, 75] which fall under technological strategies.

**Table 5:** Identified strategy of cost awareness (*n* = 54)

Strategy		Cites	Number of studies (total n = 54)	% of the total number of studies
A. Human resources	Cost education	[17, 19, 21, 22, 28, 29, 44–56, 59, 61, 63–70, 72–74, 78, 80, 82, 83, 85–88]	41	75,9
	Staff collaboration	[46, 47, 61, 66, 68, 73, 82]	7	12,9
	Incentive program	[22, 55, 57, 58, 60, 63, 64, 66, 71, 77, 78, 88]	12	22,2
B. Organizational strategy	Cost containment policy	[2, 22, 48–54, 58, 63, 67, 72]	13	24,1
	Standardization program	[27, 49, 50, 72, 73, 82]	6	11,1
	Mangement support	[55, 71, 84]	3	5,6
C. Technology	Visual pricing	[2, 19, 21, 28, 43, 45, 47–51, 54–56, 61, 64, 68, 69, 71, 72, 78, 84, 86, 87]	23	42,6
	Accounting information system	[28, 43, 55, 56, 59, 64–66, 73, 75, 81–84, 87]	15	27,8
D. External relationship	Regulation	[28, 29, 57, 80, 85]	5	9,3
	Patient-provider interaction	[27, 29, 47, 52, 60, 82, 83]	6	11,1
	Supplier-provider interaction	[28, 47, 80, 85]	4	7,4
	Academic curriculum	[51, 57, 64, 76, 81, 82, 84]	7	12,9

### Human resources strategy

Cost education was most common in human resource strategy. The content of education covers imparting knowledge on effective cost management [47], knowledge of costs [49, 51, 52, 63], evidence-based decision-making [69, 87], high-value services [72, 73], cost standardization [66], reimbursement processes [50], the actual costs of commonly used healthcare materials [75, 76] and waste reduction [74]. Several trials [44, 58, 81] and intervention studies [64, 67, 87] conducted in the US and Europe demonstrated the effectiveness of cost education for doctors, residents, and specialists to improve cost awareness, cost efficiency, and cost-effectiveness. In addition to increased awareness, cost education improved efficiency through cost reduction, such as disposable instrument expenses [87], diagnostic test costs [58], and expenditures in inpatient care [44], obstetrics and gynecology units [81], and intensive care units [67]. Furthermore, cost-effectiveness was improved through shorter lengths of stay (LOS) [44, 88], reduced operating times [87], and lower rates of mortality and cardiac arrest [67]. The process of delivering cost education was not a one-time event, with regular feedback mechanisms in place [85].

Less common strategies were incentive programs and staff collaboration. Rewards and punishments [44, 86] were linked to the use of cost-effective equipment and medications [27, 75], investment in patient education [62], standardization of supply chains [88] and prescribing practices [57]. Staff collaboration among the healthcare professionals [61, 73] or between healthcare professionals and management [47, 82] was also applied. Doctors could play a significant role as role models in promoting cost awareness practices [68, 77].

### Organizational strategy

Three organizational strategies were reported, i.e. cost containment, standardization program, and securing management support. Cost containment is focused on helping promote cost awareness through measures such as informative campaigns [22], resource restriction policies [54], and cost-effective practices like financial audits [63, 67]. Standardizing programs across organizations can be achieved by implementing uniform clinical guidelines [72] and equipment [82], thus ensuring consistency in care delivery and resource utilization. Finally, securing management support to address cost-related issues [62] and financial support [66] is vital for the successful implementation of cost management strategies [84].

### Technology strategy

Technology-driven strategies, such as visual pricing systems and software tools, are essential for improving financial transparency by providing clear cost information to healthcare providers and patients [19, 72, 87]. Three trials [43, 45, 86] and one intervention [48] conducted in the US showed that visual pricing systems significantly reduced cost [86], laboratory test request [43], and muscle relaxant drug expenses [45], particularly the high-cost drug [48]. Other variables such as complication rates, length of stay (LOS), or mortality [86]; and admission rates, emergency department visits, or outpatient follow-up visits were not affected [43]. Accounting information systems are crucial for tracking and managing costs [22, 55], helping establish structured cost-management practices [75], and ensuring optimized resource utilization across healthcare services [83].

### External relationship strategy

Academic curricula and patient-provider interactions were frequently mentioned, suggesting their impact on aligning professional practices with cost-awareness principles. Engaging with academic institutions to embed cost-awareness topics in medical education [38, 51] and developing closer relationships with patients through effective communication [29, 60] created opportunities to foster cost awarenes. Similarly, regulation and supplier-provider interactions, albeit to a lesser extent. Cost containment policies [57] and collaborative programs [28, 80] from regulatory bodies help support cost awareness initiatives. Additionally, fostering relationships with suppliers [85] and adopting joint purchasing patterns with other hospitals [29, 60] enhanced cost-awareness efforts.

**Fig. 2:**
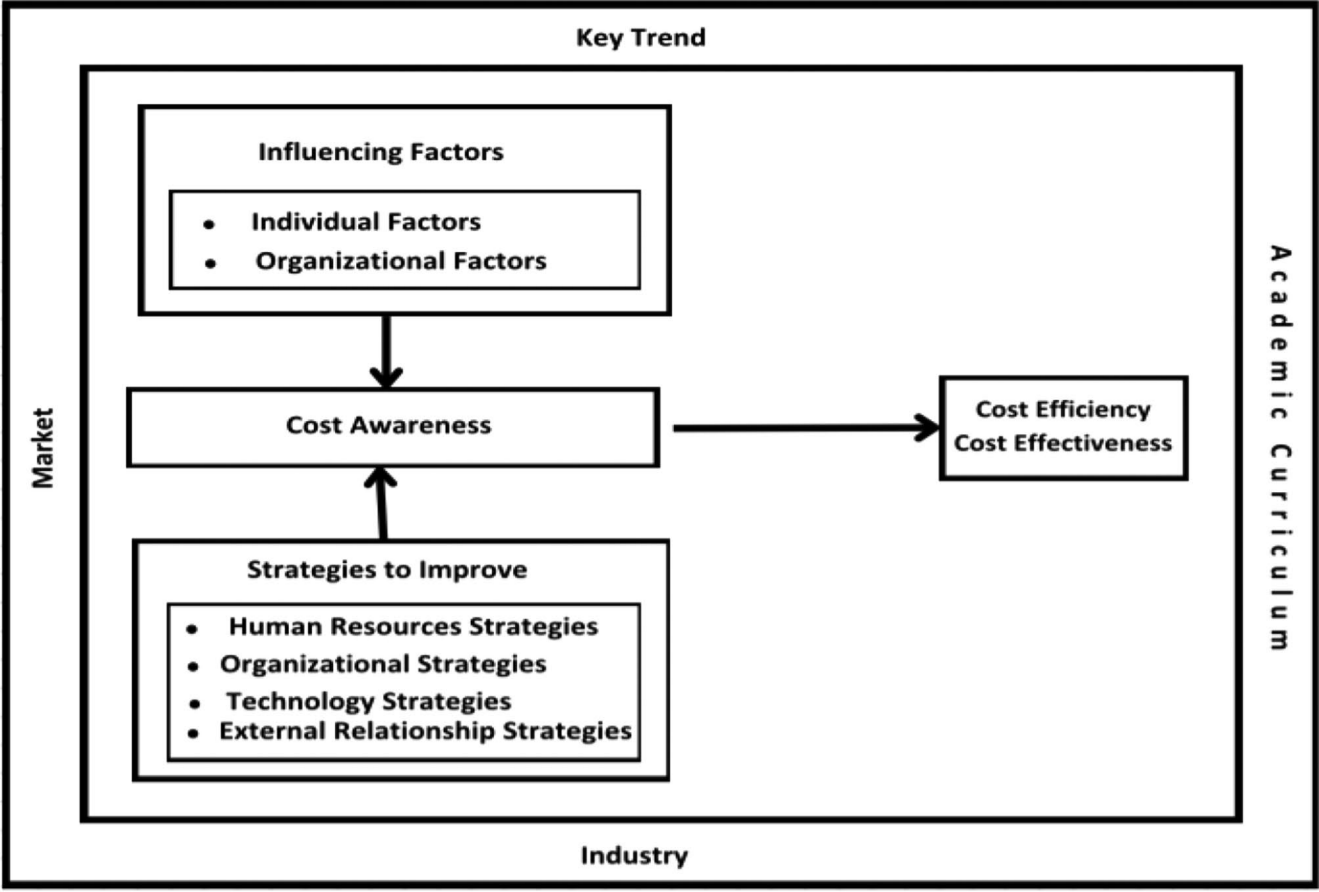
A conceptual framework on cost awareness

Figure 2 demonstrates internal and external factors affecting cost awareness within hospitals and four strategies to improve cost awareness. Internal factors are depicted within the hospital environment (inside the diagram), consisting of individual and organizational factors; while external factors are shown outside the hospital environment (i.e. key trend factors, market, industry, and academic factors). To improve cost awareness, four strategies are identified, i.e. human resource, organizational, technological, and external relations strategies. The strategies are placed within the hospital environment as there are initiated and developed by the hospital. By integrating these strategies, healthcare institutions can foster a culture of financial responsibility, ultimately improving resource utilization and ensuring sustainability of healthcare services in diverse settings

## Discussion

In this review, we categorized the influencing factors into internal and external factors. Internal factors refer to characteristics or conditions that exist within the hospital setting, while external factors are broader systemic or societal factors from outside the hospital.

The scoping review highlights that internal factors, including professional knowledge, experience, and organizational support, are the most significant determinants of cost awareness among hospital workers, while external factors such as healthcare financing policies, patient expectations, and academic initiatives also play a vital role, albeit to a lesser extent. The most frequently cited and applied strategies for enhancing cost awareness are human resource-focused initiatives, followed by technological tools, organizational policies, and external relationships. These findings underscore that limited professional knowledge [21, 45, 47, 49, 65], coupled with a lack of access to price information [45, 56], creates systemic barriers to effective cost-awareness practices. Low-cost knowledge is due to the lack of cost education in the medical curriculum [72], the absence of cost education mandates in employee recruitment processes [74], and the low quality of cost education provided within hospitals [62, 69, 72]. A lack of access to price information impedes economic decision-making [28, 57], particularly regarding diagnostic test ordering [81], which can consequently burden patients with excessive expenses. Yet, not all tests and medications are deemed essential [19]. Research conducted in China indicates that while most doctors recognize their important role in medical resource allocation and cost management, they struggle with implementation due to the absence of comprehensive price information [73]. Studies also show that doctors in maritime countries such as Australia and New Zealand tend to have higher cost awareness, potentially due to more structured financing systems and institutional norms [19]. Meanwhile, those working in primary care—often experiencing high workloads and burnout—may exhibit lower cost attentiveness [59]. Interestingly, no significant difference was observed between physicians in urban and rural settings [60], indicating that institutional culture and training may have a greater influence on cost awareness than geographic location alone.

These findings infer that cost awareness is a multi-dimensional issue, where individual education and visual pricing are critical entry points for improvement. Lack of professional knowledge on healthcare costs serves as a major barrier, while structured educational programs and transparent access to pricing information emerge as facilitators. Education programs demonstrated significant benefits. Several trials [44, 58, 81] and intervention studies [64, 67, 87] conducted in the United States and Europe have shown its effectiveness in reducing costs, such as disposable instrument expenses [87], diagnostic test costs [58], and expenditures in inpatient care [44], obstetrics and gynecology units [81], and intensive care units [67]. Furthermore, cost education has been linked to improved outcomes, including shorter lengths of stay (LOS) [44, 87], reduced operating times [87], and lower rates of mortality and cardiac arrest [67]. Visual pricing systems were effective in reducing healthcare expenses and fostering transparency [19, 43, 86]. Trials conducted in the US [43, 45, 86] and one intervention study [48] demonstrated significant reductions in costs, including laboratory test requests [43], anesthetic agent expenses [45], and high-cost drug utilization [48]. Other variables such as complication rates, LOS, or mortality [86]; and admission rates, emergency department visits, or outpatient follow-up visits were similar [43].

This scoping review builds upon earlier studies, particularly two systematic reviews (published in the years 2007 and 2008) and one scoping review (published in 2024). Unlike those reviews that focused on physicians [89–91], this scoping review includes all hospital workers, such as nurses and administration staff to map and summarize factors affecting cost awareness among them. By considering a broader target population and extending the time period for review (from 1990 to March 2024), this review provides an updated perspective compared to earlier works. The previous systematic reviews covered research only until May 2005 [89, 90], and the earlier scoping review encompassed studies from 2015 to 2021 [91]. Therefore, this expanded scoping review is expected to provide strategies informed by the latest findings relevant to cost awareness among diverse hospital workers. Additionally, we identify key strategies for improving cost awareness, categorized into human resources, technological, organizational, and external relationship strategies, based on innovation cost management theory [46]. The specificity and granularity of this categorization provide a structured roadmap for future interventions, surpassing the scope of previous reviews.

### Knowledge gaps and implications

The findings of this scoping review underscore the critical role of cost awareness in hospitals, serving as a key strategy to alleviate financial burdens on healthcare systems and ensure the sustainability of service delivery [73]. Strengthening cost awareness has gained increasing attention in both healthcare management and medical education, spanning undergraduate and postgraduate curricula [84]. However, significant challenges remain in translating cost awareness strategies into practice. These challenges arise from multiple barriers including: (a) doctors’ concerns about malpractice lawsuits, which may deter cost-conscious decision-making [54]; (b) doctors’ limited confidence in improving cost awareness due to the centralized nature of cost regulation at governmental and institutional levels, leaving them with little autonomy in financial decision-making; and (c) the generally low level of public awareness regarding healthcare costs [27], which can lead to tensions between doctors and patients when discussing treatment options. Addressing these barriers requires multifaceted [85] and multisectoral [57] strategies that involve individual, organizational, and policy-level interventions. A multifaceted approach involves addressing multiple factors that influence cost consciousness, such as education, incentives, and pricing transparency [85]. Meanwhile, a multisectoral approach requires collaboration among policymakers, hospital administrators, educators, and healthcare professionals to ensure that cost awareness initiatives are effectively implemented and sustained [57].

Additionally, an important geographic disparity was identified in the existing literature, with most studies conducted in the US (29 studies, 53.7%) and Europe (14 studies, 25.9%). Research on cost awareness in Asia (7 studies, 13%) and Africa (3 studies,5%) remains notably scarce [46, 54, 56, 63, 72, 73, 77, 84, 85, 87]. In the US, the financing structures and high resources support cost education and integrated decision-support tools, such as through advanced technological systems [43, 44, 48, 51, 52, 55, 58, 60, 64, 67–71, 74–76, 78–83], while the European studies reflect a more centralized health system and emphasize policy-driven cost containment through institutional reforms and regulatory mechanisms [49, 50, 53, 57, 59, 61, 62, 66, 86, 88]. In contrast, systemic challenges such as financial constraints, weak institutional capacity, hierarchical organizational cultures, and lack of leadership support were highlighted from studies in Asia and Africa settings [46, 54, 56, 63, 72, 73, 77, 84, 85, 87]. These differences limit the generalizability of the existing cost awareness strategies to the more resource-constrained setting in the LMIC contexts. Hence, future research on developing and validating cost-awareness interventions tailored to the unique financial, cultural, and institutional realities in the LMIC settings is needed [92–94].

Differences in health system models between developed and developing regions may shape cost awareness dynamics in distinct ways. For instance, the market-driven nature of the U.S. healthcare system—characterized by high out-of-pocket payments and private insurance—places greater emphasis on financial accountability at the provider level, potentially fostering a stronger culture of cost awareness [95, 96]. In contrast, the Bismarck model (common in Germany and France) and the Beveridge model (used in the U.K. and Nordic countries) operate under more centralized cost-control mechanisms, where cost-awareness efforts may be more institutionally driven rather than reliant on individual healthcare providers [97]. Although this study did not conduct a direct comparative analysis, recognizing these systemic differences provides valuable context for interpreting cost-awareness trends across regions.

### Limitation of the study

The scope of the literature search was limited to hospital settings, overlooking other contexts relevant to efforts aimed at mitigating healthcare cost burdens, such as clinics, community health centers, and private practitioners. Consequently, there is a need for further investigation to assess cost awareness in healthcare services beyond hospital environments.

Secondly, the study focused solely on hospital workers as the study population. However, cost awareness is influenced by both internal and external factors. Thus, additional research involving patients, government officials, insurance providers, and medical students, is essential to provide a more comprehensive understanding of cost awareness dynamics.

Thirdly, while papers on value-based care were excluded, this decision was made to maintain conceptual clarity, as value-based care encompasses a broad range of financial and quality-related strategies beyond the specific scope of cost awareness. However, we acknowledge that aspects of value-based care, such as provider incentives and efficiency measures, may intersect with cost awareness initiatives.

Fourth, this review did not include a formal quality appraisal of the included studies as this was not mandatory in scoping review methodology. This may limit the ability to assess the robustness of the available evidence.

Finally, the inclusion of English-language sources may have restricted important studies from non-English-speaking regions, particularly in Asia and Africa, where healthcare financing challenges differ from those in high-income countries. This limitation underscores the need for multilingual research efforts to capture a more diverse range of perspectives.

## Conclusion

This review highlights the complexity of cost awareness in hospitals, which is influenced by a combination of internal and external factors, with professional knowledge, experience, and access to price information identified as key determinants. Cost education and visual pricing systems were the most reported strategies, predominantly implemented in high-income contexts. These findings highlight the need for future research on developing and testing context-specific—particularly in low- and middle-income countries—, multifaceted interventions to strengthen cost awareness across diverse hospital settings.

## Electronic supplementary material

Below is the link to the electronic supplementary material.

Supplementary Material 1: PRISMA Scoping Review Checklist

## Data Availability

Data is provided within the manuscript.

## Abbreviations

DRG: Diagnosis Related Group
FGD: Focus Group Discussions
GDP: Gross Domestic Product
LMICs: low- and middle-income countries
LOS: Length of Stay
PCC: Population, Concept, and Context
PRISMA-ScR: Preferred Reporting Items for Systemic reviews and Meta-Analyses extension for Scoping Reviews
P4P: pay-for-performance
WHO: World Health Organization
